# Anastomotic leakage prevention using dry-preserved fibroblast cell sheets in esophageal reconstruction

**DOI:** 10.1016/j.reth.2025.09.011

**Published:** 2025-09-30

**Authors:** Hiroshi Kurazumi, Ryunosuke Sakamoto, Koji Ueno, Akihiro Fujita, Kazumasa Matsunaga, Masashi Yanagihara, Yoshihiro Takemoto, Junichi Murakami, Atsunori Oga, Shunsaku Katsura, Kimikazu Hamano

**Affiliations:** a Department of Surgery and Clinical Science, Yamaguchi University Graduate School of Medicine, Ube, Yamaguchi, Japan; b Department of Tissue Regenerative Medicine, Faculty of Medicine and Health Sciences, Yamaguchi University, Ube, Yamaguchi, Japan; c Division of Advanced Cell Therapy, Research Institute for Cell Design Medical Science, Yamaguchi University, Ube, Yamaguchi, Japan; d Department of Clinical Laboratory and Biomedical Sciences, Division of Health Sciences, Graduate School of Medicine, The University of Osaka, Suita, Osaka, Japan; e Department of Molecular Pathology, Yamaguchi University Graduate School of Medicine, Ube, Yamaguchi, Japan; f Department of Medical Education, Yamaguchi University Graduate School of Medicine, Ube, Yamaguchi, Japan

**Keywords:** Dry-preserved fibroblast sheets, Wound healing, Esophageal anastomotic leakage, Collagen, Growth factor

## Abstract

**Background:**

Anastomotic leakage is a common and serious complication of esophageal reconstruction, and new methods are required for its prevention in clinical settings. We herein developed dry-preserved fibroblast cell sheets (dry sheets), which are easy to use and promote wound healing. This study aimed to investigate the efficacy of transplanting allogeneic dry sheets in preventing anastomotic leakage in a rat esophageal reconstruction model.

**Methods:**

Allogeneic dry sheets were prepared from the rat oral mucosa. A rat esophageal anastomosis model was created, and two dry sheets were applied to cover the anastomotic sites. Anastomotic leakage incidence, burst pressure, histological findings, and collagen contents were compared between the control and dry sheet groups postoperatively.

**Results:**

The dry sheet group demonstrated a lower incidence of anastomotic leakage than the control group (control: 64 % vs. dry sheet: 28 % on day 3, control: 57 % vs. dry sheet: 29 % on day 5). Abscess scores at the esophageal anastomotic sites were also lower in the dry sheet group than in the control group on days 3 and 5. Burst pressure was significantly higher in the dry sheet group than in the control group on days 3 and 5. Collagen type I was significantly increased in the dry sheet group compared with that in the control group.

**Conclusions:**

Allogeneic dry sheet application improved anastomotic leakage incidence and burst pressure, indicating the usefulness of these sheets in preventing esophageal anastomotic leakage.

## Highlights

• Allogeneic dry-preserved fibroblast cell sheets reduce anastomotic leakage rate.
• Dry-preserved fibroblast cell sheets release various growth factors.
• Dry-preserved fibroblast cell sheets help in collagen production.

## 1 Introduction

Anastomotic leakage is a prevalent complication of gastrointestinal surgery, posing a significant challenge. In particular, esophageal anastomotic leakage is reported in 11.4 %–21.2 % of esophageal reconstruction cases. This complication frequently requires reoperation, which prolongs hospitalization and increases medical expenses [1]. Covering the anastomosis with autologous tissues, such as pleura, pericardial fat, and omentum, has been explored as a prevention strategy [2,3]. However, current techniques have not eliminated anastomotic leakage.

To promote the widespread adoption of regenerative medicine, it is not only important to establish the safety and efficacy of treatments but also to reduce their costs. Therefore, we focused our research on fibroblast-based cell sheets [4]. Initially, we fabricated autologous fibroblast cell sheets, applied them to skin ulcers, and confirmed their therapeutic efficacy [[4], [5], [6]]. Based on these findings, we conducted a clinical study that involved six patients with intractable venous ulcers [7]. Three patients achieved complete healing following autologous cell sheet transplantation. The remaining three patients did not undergo transplantation due to the inability to obtain high-quality autologous fibroblasts that met the standards. This limitation led us to investigate the use of allogeneic cells instead of autologous cells.

In a murine skin defect model, we demonstrated that allogeneic fibroblast sheets promote wound healing to a comparable extent as autologous cell sheets [8]. We also hypothesized that these cell sheets may be effective in preventing postoperative complications beyond skin ulcers. Accordingly, we assessed their efficacy in esophageal reconstruction [9], pancreatic fistula [10], and bronchial leakage models [11], revealing that the cell sheets effectively reduced the incidence of such complications in all models.

To further reduce costs, we developed a cryopreservation method for these cell sheets, which was confirmed to be effective [12]. Conversely, problems related to cost and facilities, including cold-chain storage and transportation, persist. Considering the broader clinical applicability, dry preservation of cell sheets may provide advantages over living cell sheets in terms of convenience and cost-effectiveness. We previously demonstrated allogeneic dry-preserved fibroblast cell sheets (dry sheets) in a full-thickness skin defect model of diabetic mice through a novel mechanism of releasing intracellular growth factors by rehydration [13]. In the present study, we aimed to investigate the effectiveness of allogeneic dry sheets in preventing anastomotic leakage in an esophageal reconstruction model.

## 2 Materials and methods

### 2.1 Animals

Japan SLC (Shizuoka, Japan) supplied male Wistar/ST and SD rats (aged 10 weeks). The rats were housed in a controlled environment with a temperature of 22 °C ± 2 °C, humidity of 70 % ± 20 %, and a 12-h light/dark cycle. They had access to food and water *ad libitum* preoperatively, and only water was allowed postoperatively. This study was conducted in accordance with all relevant guidelines and was approved by the Institutional Animal Care and Use Committee of Yamaguchi University (IACUC; No. 31-008).

### 2.2 Preparation of dry-preserved multilayered fibroblast sheets

Multilayered fibroblast cell sheets (living sheets) were made using a previously reported method [6,10]. In brief, male SD rats (aged 10 weeks) were anesthetized with 5 % isoflurane (MSD Animal Health, Tokyo, Japan), and oral mucosal tissue was collected. It was minced and incubated in Dulbecco's Modified Eagle's Medium (Thermo Fisher Scientific, Waltham, Massachusetts, USA) supplemented with 10 % fetal bovine serum (FBS; Thermo Fisher Scientific, Waltham, Massachusetts, USA), 5 % collagenase (FUJIFILM Wako, Osaka, Japan), and 1 % penicillin–streptomycin (Thermo Fisher Scientific, Waltham, Massachusetts, USA) at 37 °C in a humidified incubator containing 5 % CO_2_. On the following day, the culture medium was centrifuged, and the supernatant was removed. The remaining tissue fragments were cultured in the medium for 3 days. The culture medium was collected using 0.05 % trypsin-ethylenediaminetetraacetic acid (Thermo Fisher Scientific, Waltham, Massachusetts, USA) and passed through a 40 μm cell strainer to remove the tissue fragments. The culture medium was centrifuged again, and the supernatant was extracted. The residual cells were then cultured in the medium for 4 days. A total of 5.0 × 10^5^ cells were seeded into each well of a 24-well plate with 2 mL of medium. On the following day, the culture medium was replaced with 2 mL of CTS™ AIM V™ SFM (Thermo Fisher Scientific, Waltham, Massachusetts, USA) and HFDM-1 (+) (Cell Science & Technology Institute, Miyagi, Japan) supplemented with 5 % FBS. After 24 h, the culture medium was collected before the detachment of cell sheets and was stored at −80 °C until the growth factors were measured. After washing with phosphate-buffered saline (PBS), each fibroblast cell sheet was incubated with 500 μL of dispase solution (10 PU/mL, FUJIFILM Wako) for 1 h. After washing twice with PBS, multilayered fibroblast sheets (living sheets) were gently detached from the culture plates. These living sheets were transferred onto a silicon pedestal, and air-drying was performed inside a bioclean bench to prepare the dry-preserved cell sheets (dry sheets), as previously described [13,14]. These dry fibroblast sheets were stored in a refrigerator (4 °C) for 1 week to 1 month before use.

### 2.3 Esophageal anastomosis model

The esophageal anastomosis model was prepared as previously described [9]. In brief, Wistar/ST rats were anesthetized with 5 % isoflurane and positioned supine with neck extension. The anesthesia concentration was reduced to 2 % after the initial 5 min. The neck was shaved, and a 2-cm longitudinal incision was made. The trachea and esophagus were separated. The esophagus was pulled in front of the trachea and fixed with a clip. It was then transected, and the stump was sutured with three stitches using a 7-0 polypropylene thread (Medtronic, Minneapolis, Minnesota, USA). The esophagus was repositioned behind the trachea, and the wound was closed. All procedures were performed by the same surgeon (R.S.).

### 2.4 Implantation of dry-preserved multilayered fibroblast sheets

Two dry sheets were applied with one sheet placed on the posterior wall and the other on the anterior wall. Both dry sheets were extended to the lateral walls to ensure complete coverage of the anastomosis circumference.

### 2.5 Assessment of anastomotic leakage and pressure resistance

The rats were sacrificed on postoperative days 3 and 5. Anastomotic leakage was defined as the presence of an abscess around the anastomosis site. The severity of the abscess was graded as follows; 0 (no abscess), 0.5 (one small abscess [<1 mm]), 1 (several small abscesses), 2 (one moderate abscess [1–3 mm]), 3 (one large abscess [3–5 mm] or several moderate abscesses), and 4 (one very large abscess [>5 mm] or several large abscesses) [15,16].

The esophagus was excised from the glottis level to the abdominal esophagus. The 24-G catheter was inserted from the anal side of the esophagus. Both ends of the esophagus were ligated with 4-0 nylon sutures (Nitcho Kogyo Co., Ltd., Tokyo, Japan). The 24-G catheter was connected to an aneroid sphygmomanometer (HT-1500, Nihon Seimitsu Sokki Co., Ltd., Gunma, Japan) for anastomotic pressure resistance measurement. The esophagus was submerged in water, and air was injected until leakage was detected from the anastomosis site. The pressure at which the first leak occurred was recorded as the burst pressure [9].

### 2.6 Histological analysis of the cell sheets and esophageal anastomosis site

Living and dry fibroblast sheets were applied to the ham tissue and subsequently fixed in 10 % neutral-buffered formalin (Fujifilm Wako) for histological specimen preparation. The rats were sacrificed on postoperative days 3 and 5, and their esophagi were excised and fixed in 10 % neutral-buffered formalin for histological examination. The specimens were stained with hematoxylin–eosin (HE) and azocarmine aniline blue (Azan).

All histological analyses were conducted in a blinded manner and reviewed by a pathologist (A.O.).

### 2.7 Measurement of growth factors eluted from the cell sheets into the medium

Each dry sheet was immersed in a 24-well plate with 2 mL of CTS™ AIM V™ SFM and HFDM-1 (+) supplemented with 5 % FBS for 24 h at 37 °C in a humidified incubator with 5 % CO_2_. The culture medium was then collected and stored at −80 °C until measurement [13]. The concentrations of vascular endothelial growth factor (VEGF), hepatocyte growth factor (HGF), fibroblast growth factor 2(FGF2/basic FGF), and high mobility group box 1 (HMGB1) in the supernatant were measured with Quantikine Immunoassay Kits (R&D Systems, Minneapolis, Minnesota, USA) and the HMGB1 ELISA Kit Exp (SHINO-TEST CORPORATION, Kanagawa, Japan).

### 2.8 Analysis of mRNA expression levels and type I collagen content at the esophageal anastomosis site

On postoperative day 5, the rats were sacrificed, and the esophagus was excised. A 10-mm tissue segment, spanning 5 mm above and below the anastomosis site, was collected for analysis. Ribonucleic acid (RNA) was extracted using the RNeasy Mini Kit (QIAGEN N.V., Venlo, Netherlands). The total RNA concentration was quantified with a NanoDrop (Thermo Fisher Scientific, Waltham, Massachusetts, USA), and cDNA synthesis was performed with the PrimeScript™ RT reagent kits (Perfect Real Time; TaKaRa Bio Inc., Shiga, Japan). Quantitative real time polymerase chain reaction (PCR) was conducted using the QuantStudio™ 3 Real Time PCR system (Thermo Fisher Scientific, Waltham, Massachusetts, USA) and SYBR™ Select Master Mix (Thermo Fisher Scientific, Waltham, Massachusetts, USA). Table 1 summarizes the primer sequences used in the present study. Actin beta served as an endogenous control, and mRNA expression levels were estimated using the ΔCT method. Collagen quantification was conducted using the Rat Type I Collagen Detection Kit (Chondrex, Inc, Redmond, Washington, USA).

**Table 1:** Polymerase chain reaction primer sequences.

	Forward	Reverse
ACTB	CTACCTCATGAAGATCCTGACCGAG	TTTCCCTCTCAGCTGTGGTGG
Collagen type I	ATGCTGCCTTTTCTGTTCCTTTCTC	TTTGGGGAGCAATGGAGGAGAG
Collagen type III	CACCCTGAACTCAAGAGCGGA	CATCCATCTTGCAGCCTTGGTTAG

### 2.9 Statistical analysis

Continuous variables are expressed as mean and standard deviation for normally distributed data and as median with interquartile range for non-normally distributed data. Differences between groups were analyzed using the Student's *t*-test or Mann–Whitney *U* test, as appropriate. A *p*-value of <0.05 was considered statistically significant. Statistical analyses were conducted using STATA17 (StataCorp LLC, College Station, Texas, USA).

## 3 Results

### 3.1 Histological analysis of the living and dry-preserved fibroblast sheets

**Fig. 1:**
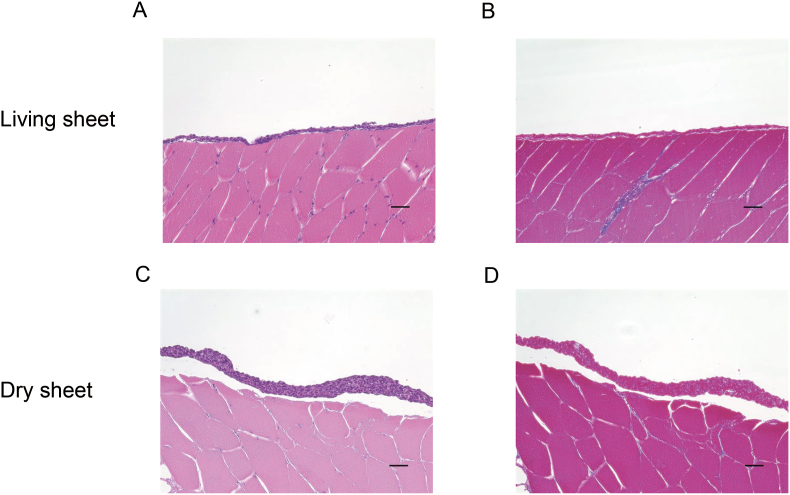
Pathological analysis of the sections of the living fibroblast sheet and the dry fibroblast sheet. (A) and (B) HE staining and Azan staining in the living multilayered fibroblast sheets. (C) and (D) HE and Azan staining in the dry multilayered fibroblast sheets. Fibroblasts were isolated from the rat oral tissue. HE: hematoxylin–eosin; Azan: azocarmine, aniline blue, and orange G. The bar represents a length of 50 μm.

Cross sections of living and dry-preserved multilayered fibroblast sheets stained with HE and Azan (Fig. 1). The living fibroblast sheets demonstrated a multilayered structure with a thickness of approximately 20–30 μm, consisting of 2–3 layers of fibroblasts (Fig. 1A and B). The dry fibroblast sheets showed a multilayered structure with a thickness of approximately 40–50 μm, consisting of 4–5 layers of fibroblasts (Fig. 1C and D). The dry sheets were slightly thicker than the living sheets, and they shrank and became more variable in size when dried.

### 3.2 Measurement of growth factors released from the cell sheets

**Fig. 2:**
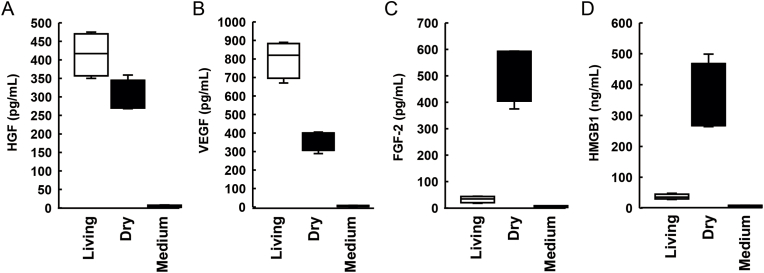
Comparison of growth factors and cytokines released from living and dry sheets into the medium. (A) HGF, (B) VEGF, (C) FGF-2, and (D) HMGB1. The *n* number for each group is 6.

The growth factors and cytokines released from the living and dry-preserved sheets into the medium were measured. The HGF concentration was 442 ± 63 pg/mL in living sheets and 309 ± 46 pg/mL in dry sheets. The VEGF concentration was 810 ± 82 pg/mL in living sheets and 398 ± 71 pg/mL in dry sheets. The FGF-2 concentration was 26 ± 15 pg/mL in living sheets and 519 ± 88 pg/mL in dry sheets. The HMGB-1 concentration was 30 ± 12 ng/mL in living sheets and 349 ± 89 ng/mL in dry sheets. HGF and VEGF were detected in both cell sheets; however, FGF-2 and HMGB-1 levels were more than 10-fold higher in the dry fibroblast sheets than in the living fibroblast sheets (Fig. 2).

### 3.3 Histological analysis

**Fig. 3:**
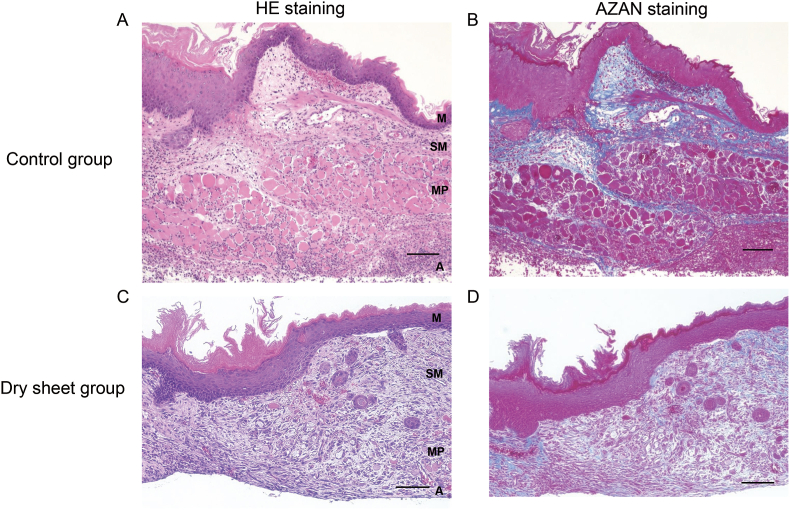
Representative histological images of HE and Azan staining of the esophageal anastomotic sites on postoperative day 5. (A) and (B) HE and Azan staining in the control group. (C) and (D) HE and Azan staining in dry fibroblast sheets. The bar represents a length of 100 μm. M: mucosal layer; SM: submucosa; MP: muscularis propria; A: adventitia.

Fig. 3 illustrates representative images of HE and Azan staining on postoperative day 5. The mucosal epithelium was completely re-epithelialized in both groups; however, the muscular layer remained separated. In the control group, numerous inflammatory cells were observed in the submucosa, with minimal evidence of esophageal glands and neovascularization. In contrast, the dry sheet group showed infiltration of inflammatory cells, along with the partial presence of esophageal glands in some areas (Fig. 3A and C). The area of the collagen fibers, as stained by Azan, appeared slightly greater in the dry sheet group (Fig. 3B and D).

### 3.4 Incidence of anastomotic leakage

The incidence of anastomotic leakage incidence was 64 % and 28 % of 14 on postoperative day 3 and 57 % and 29 % on postoperative day 5 in the control and dry sheet groups, respectively. Anastomotic leakage incidence was not significantly lower in the dry sheet group than in the control group on both postoperative days 3 (*p* = 0.06) and 5 (*p* = 0.07). However, anastomotic leakage incidence was lower in the dry sheet group than in the control group. No mortality was observed in either group on postoperative days 3 and 5 (Table 2).

**Table 2:** Anastomotic leakage incidence and mortality rates.

	Day 3			Day 5		
	Control	Dry sheet	*p*-value	Control	Dry sheet	*p*-value
Anastomotic leakage	9/14 (64 %)	4/14 (28 %)	0.06	8/14 (57 %)	4/14 (29 %)	0.07
Mortality	0/14	0/14	–	0/14	0/14	–

### 3.5 Abscess score at the anastomotic site

**Fig. 4:**
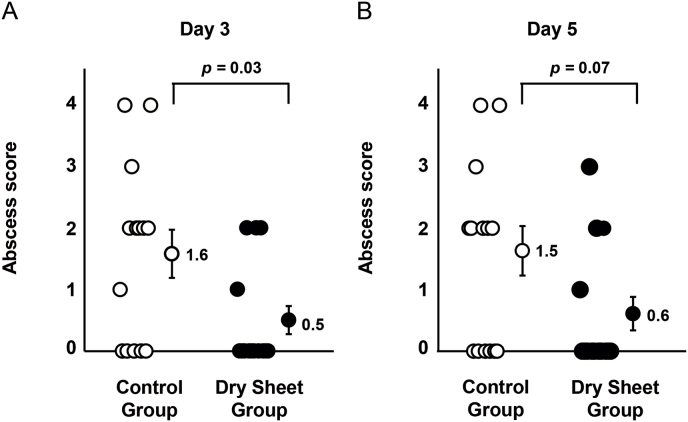
Suture assessment based on abscess scores. (A) abscess score on postoperative day 3 [5]. Abscess score on postoperative day 5. The *n* number for each group is 14.

The mean abscess scores were 1.6 and 0.5 on postoperative day 3 and 1.5 and 0.6 on postoperative day 5 in the control and dry sheet groups, respectively. The abscess score was significantly lower in the dry sheet group compared to the control group on postoperative day 3 (*p* = 0.03) (Fig. 4A). Although the difference in abscess scores between the two groups on postoperative day 5 was not statistically significant (*p* = 0.07), the dry sheet group showed a noticeably lower score than the control group (Fig. 4B).

### 3.6 Burst pressure at the anastomotic sites in both groups

**Fig. 5:**
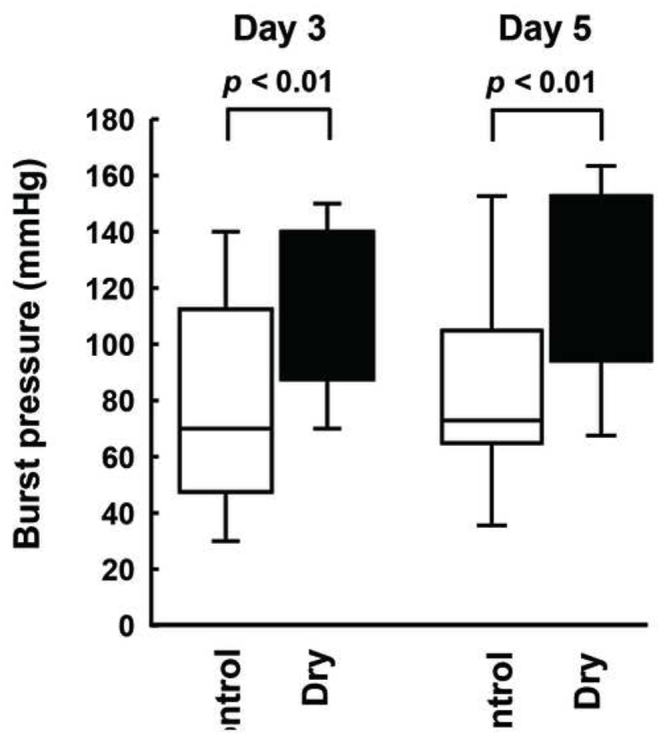
The burst pressure at the anastomotic site on postoperative days 3 and 5. The *n* number for each group is 14.

The burst pressure resistance at the anastomotic site was 79 ± 35 mmHg and 121 ± 28 mmHg on postoperative day 3 and 86 ± 31 mmHg and 124 ± 30 mmHg on postoperative day 5 in the control and dry sheet groups, respectively. The pressure resistance was significantly higher in the dry sheet group compared to the control group on both postoperative days 3 (*p* < 0.01) and 5 (*p* < 0.01) (Fig. 5).

### 3.7 Analysis of collagen mRNA and protein expression levels at the anastomotic sites

**Fig. 6:**
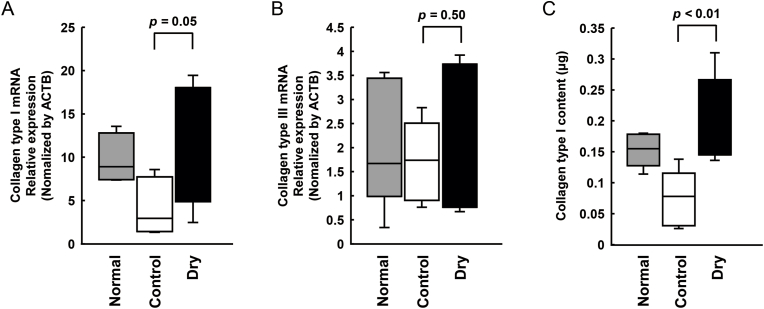
Analysis of collagen expression levels at the anastomotic site on postoperative day 5. (A) and (B) Collagen type I and type III mRNA relative expression. (C) Assessment of collagen type I level. Normal: normal esophageal tissue from Wister/SD rats at the same weekly age. The *n* number for each group is 6.

The dry sheet group showed higher collagen type I mRNA expression compared to the control group (*p* = 0.05) on postoperative day 5 (Fig. 6A). Further, no significant difference in the expression level of collagen type III mRNA was found among the three groups on postoperative day 5 (Fig. 6B). The collagen levels were 0.08 ± 0.04 μg and 0.19 ± 0.07 μg in the control and dry sheet groups, respectively, indicating a significantly higher collagen type I level in the dry sheet group (*p* < 0.01) (Fig. 6C).

## 4 Discussion

In this study, we revealed that the application of allogeneic dry sheets reinforces anastomotic tissue and prevents anastomotic leakage in a rat esophageal reconstruction model. The dry sheet group demonstrated significantly higher burst pressure than the no-treatment group on postoperative days 3 and 5 (Fig. 5). In addition, the dry sheet group showed significantly lower abscess scores on postoperative day 3 (Fig. 4). Conversely, anastomotic leakage incidence was lower in the dry sheet group than in the control group on postoperative days 3 and 5 but with no significant difference between them (Table 2). A statistically significant difference in anastomotic leakage incidence could be obtained using a larger number of animals. However, the effectiveness of the dry sheet was confirmed in other endpoints (burst pressure and abscess score) in this study; thus, the principle of animal welfare was observed and no further experiments that involved animal sacrifice were performed. These results indicate that allogeneic dry sheets could be used as tissue-reinforcing material in surgical procedures.

Notably, the dry sheet group demonstrated a significantly higher burst pressure and stronger tissue reinforcement than the control group (Fig. 5). This finding was supported by the significantly increased production of type I collagen at the anastomotic site in the dry sheet group (Fig. 6). Growth factors that are released from the dry sheet may induce type I collagen production. Similar to mouse and human dry sheets [13,14], rat dry sheets also release high levels of FGF2 and HMGB1, as well as VEGF and HGF, when immersed in culture medium for 24 h (Fig. 2). These findings suggest that growth factors might be released from dry sheets at an early stage following transplantation, thereby contributing to tissue repair. Previous studies have reported no metabolic activity upon re-culture of dry sheets [13]. In this study, dry sheets were stored in a refrigerator (4 °C) for at least 1 week after preparation. Therefore, it is believed that almost all cells in the dry cell sheets are non-viable at the time of transplantation. The drying process likely damages cell membranes, resulting in sheets composed primarily of dead cells. Upon rehydration, these sheets rapidly release high levels of stored intracellular growth factors [13]. Previous studies investigating the mechanism of action of mouse and human dry sheets have demonstrated that FGF2, a potent cell growth factor and tissue repair factor released by dry sheets, retains physiological activity and activates fibroblasts and vascular endothelial cells in experiments utilizing neutralizing antibodies [13,14]. Collagen production in living tissues is mainly performed by fibroblasts and is promoted by VEGF and FGF2 [17]. Therefore, it is assumed that the growth factors released following the application of the dry sheets stimulate fibroblasts in the anastomotic tissue. This promotes type I collagen production and fibroblast proliferation. In addition to these factors, HGF contributes to esophageal mucosa repair in a rat model [18] and HMGB1 induces skeletal muscle regeneration [19,20], indicating that these growth factors that are released from dry sheets act simultaneously at the suture site and contribute to tissue repair. Dry sheets are not expected to exert paracrine effects through continuous growth factor secretion, as is the case with conventional living cell sheets. Despite this, the results of this study and our previous study revealed that the dry sheet promotes angiogenesis, wound healing, and tissue reinforcement *in vivo*, indicating the importance of bioactive growth factors released from the dry sheet for its mechanism of action [13].

We have focused on fibroblasts and have researched regenerative medicine products using these cells [[4], [5], [6], [7], [8], [9], [10], [11], [12], [13],^21^,^22^]. We developed multilayered fibroblast sheets and revealed their efficacy in a skin ulcer model [6]. Subsequently, we conducted a clinical study that involved patients with intractable skin ulcers [7]. However, we encountered limitations in using autologous fibroblasts, causing us to shift our approach toward allogeneic cell application. We confirmed that allogeneic fibroblasts exhibited wound healing effects comparable to those of autologous fibroblasts in skin ulcer treatment [8]. In the future, we aim to expand the application of this cell sheet beyond intractable skin ulcer treatment to include the prevention of intestinal anastomotic leakage [9] and pancreatic fistula [10] in surgical procedures. In such cases, dry sheets are considered more practical and easier to handle than living cell sheets, particularly in laparoscopic surgery, where the ease of manipulation is essential. Accordingly, we developed dry sheets and confirmed their efficacy in a skin ulcer model [13]. When transplanting living sheets, some kind of substrate is often used to maintain the shape of the sheets. Dry cell sheets can be grasped directly with tools such as tweezers because they are dry and retain their shape. A study reported that mouse dry sheets are stable for at least 1 month [13]. In this study, rat dry sheets were produced similarly. Therefore, dry cell sheets are easy to transplant and operate. The cross-section was thicker in the dry sheets than in the living fibroblast sheet (Fig. 1). This result may be because dry sheets shrink when living fibroblast sheets dry on silicon pedestals. The ease of storage may be one of the advantages of using dry sheets.

An appropriate method for intraperitoneal delivery needs to be considered in future dry sheet applications in laparoscopic surgery. However, such delivery can be achieved using a suitable carrier material. Even a slight reduction in anastomotic leakage incidence during esophageal reconstruction would be highly beneficial not only for patients but also for healthcare providers.

In conclusion, allogeneic dry-preserved fibroblast cell sheet transplantation promotes wound healing and improves tissue strength, thereby reducing the risk of anastomotic leakage.

## Author contributions

H.K., R.S., K.U., M.Y., and K.H. contributed to the conception and designation of these experiments. H.K., R.S., A.F., K.M., and Y.T. performed the experiments. H.K., R.S., K.U., J.M., A.O., and K.H. analyzed the data. R.S., K.U., M.Y., and S.K. contributed reagents/materials/analysis tools. H.K., R.S., K.U., M.Y., and K.H. wrote the manuscript. All authors discussed the results and approved the manuscript.

## Statements and declarations

Not applicable.

## Data statement

The data that support the findings of this study are available from the corresponding author upon reasonable request.

## Declaration on generative AI and AI-assisted technologies in the writing process

The authors declare no use of generative AI and AI-assisted technologies in the writing process during the preparation of this work.

## Funding

This work was supported by a 10.13039/501100001691JSPS Grant-in-Aid for Scientific Research (C) (23K08090), the Yamaguchi prefectural subsidy program for the promotion of practical application and industrialization of regenerative medicine (Yamaguchi Prefecture Government to Central Glass Co., Ltd. and Yamaguchi University), and the Ube city subsidy program for the promotion of advanced research and development and practical application of regenerative medicine (Ube City Government to Central Glass Co., Ltd. and 10.13039/100016983Yamaguchi University).

## Declaration of competing interest

Koji Ueno and Kimikazu Hamano received a grant and consulting fees for collaborative research from Central Glass Co., Ltd. Ryunosuke Sakamoto and Akihiro Fujita were employed as research staff membersin the Joint Research Course at Yamaguchi University established by Central Glass Co., Ltd.
