# Supplementary material for: Anastomotic leakage prevention using dry-preserved fibroblast cell sheets in esophageal reconstruction
Source: Regen Ther. 2025 Sep 30;30:795–801. doi: 10.1016/j.reth.2025.09.011 (PMC12514519; doi:10.1016/j.reth.2025.09.011)
Supplement: Multimedia component 1 [file mmc1.pptx]

## Slide 1
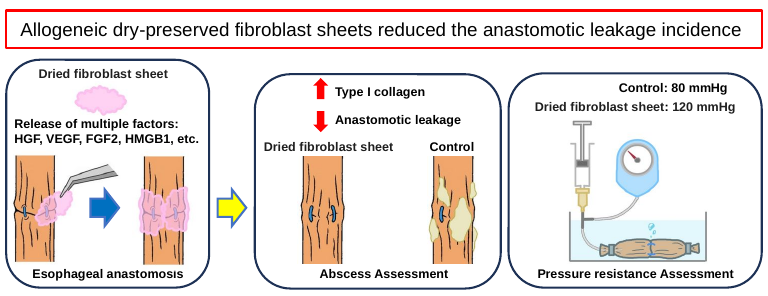

Allogeneic dry-preserved fibroblast sheets reduced the anastomotic leakage incidence
Dried fibroblast sheet
Control: 80 mmHg
Type I collagen
Dried fibroblast sheet: 120 mmHg
Anastomotic leakage
Release of multiple factors:
HGF, VEGF, FGF2, HMGB1, etc.
Dried fibroblast sheet
Control
Esophageal anastomosis
Abscess Assessment
Pressure resistance Assessment
